# Meta-barcoded evaluation of the ISO standard 11063 DNA extraction procedure to characterize soil bacterial and fungal community diversity and composition

**DOI:** 10.1111/1751-7915.12162

**Published:** 2014-09-04

**Authors:** Sebastien Terrat, Pierre Plassart, Emilie Bourgeois, Stéphanie Ferreira, Samuel Dequiedt, Nathalie Adele-Dit-De-Renseville, Philippe Lemanceau, Antonio Bispo, Abad Chabbi, Pierre-Alain Maron, Lionel Ranjard

**Affiliations:** 1 INRA, UMR1347 Agroécologie, Plateforme GenoSolDijon, France; 2 INRA, UMR1347 AgroécologieDijon, France; 3 Equipe R&D Santé-Environnement, Campus Pasteur, GenoscreenLille, France; 4 Service Agriculture et Forêt, ADEMEAngers Cedex 01, France; 5 INRA-URP3FLusignan, France

## Abstract

This study was designed to assess the influence of three soil DNA extraction procedures, namely the International Organization for Standardization (ISO-11063, GnS-GII and modified ISO procedure (ISOm), on the taxonomic diversity and composition of soil bacterial and fungal communities. The efficacy of each soil DNA extraction method was assessed on five soils, differing in their physico-chemical characteristics and land use. A meta-barcoded pyrosequencing approach targeting 16S and 18S rRNA genes was applied to characterize soil microbial communities. We first observed that the GnS-GII introduced some heterogeneity in bacterial composition between replicates. Then, although no major difference was observed between extraction procedures for soil bacterial diversity, we saw that the number of fungal genera could be underestimated by the ISO-11063. In particular, this procedure underestimated the detection in several soils of the genera *Cryptococcus*, *Pseudallescheria*, *Hypocrea* and *Plectosphaerella*, which are of ecological interest. Based on these results, we recommend using the ISOm method for studies focusing on both the bacterial and fungal communities. Indeed, the ISOm procedure provides a better evaluation of bacterial and fungal communities and is limited to the modification of the mechanical lysis step of the existing ISO-11063 standard.

## Introduction

During the last three decades, the challenge to better characterize soil microbial communities has led to the development of culture-independent techniques that are well suited to deciphering the huge diversity of soil microbes as they provide access to previously hidden genetic resources (Martin-Laurent *et al*., 2001). These methods are based essentially on the direct extraction and characterization of soil DNA. In this context, most efforts have been devoted to optimizing the soil DNA extraction procedure in order to obtain suitable representative extracts for quantitative and qualitative characterization of the microbial communities (Roesch *et al*., 2007; Rajendhran and Gunasekaran, 2008; Terrat *et al*., 2012). These efforts led to the development of various homemade DNA extraction protocols and even commercial kits (Zhou *et al*., 1996; Martin-Laurent *et al*., 2001; Delmont *et al*., 2011a; Terrat *et al*., 2012). However, each method had its own advantages and potential biases, leading to variations in DNA representativeness and consequently to effects on soil microbial assessments, making comparisons between studies impossible (Zhou *et al*., 1996; Martin-Laurent *et al*., 2001; Terrat *et al*., 2012). To deal with this issue, Delmont and colleagues (2011b) suggested that several soil sampling and DNA extraction strategies should be combined to access the whole soil microbial metagenome in terms of species richness. However, this approach is clearly not applicable or relevant to wide-scale studies, where time and cost constraints make the need to use a standardized single DNA extraction procedure obvious (Dequiedt *et al*., 2011).

In this context, a standardized ‘ISO-11063: Soil quality – Method to directly extract DNA from soil’ was developed and validated by independent laboratories to efficiently recover bacterial DNA from various soil samples (Philippot *et al*., 2010; Petric *et al*., 2011). However, archaeal and fungal groups also constitute a significant proportion of the soil microbial biodiversity and are key organisms for soil processes. In a previous study, we tested the sensitivity of the ISO-11063 method for the detection of these groups (Plassart *et al*., 2012). Briefly, three different procedures were compared on five soils with contrasting land-use and physico-chemical properties: (i) the ISO-11063 standard; (ii) a modified ISO procedure (ISOm) that includes a particular mechanical lysis step (a FastPrep®-24 lysis step instead of the recommended bead beating using a mini bead-beater cell disruptor); and (iii) a custom procedure called GnS-GII, which also includes the FastPrep®-24 mechanical lysis step. This evaluation revealed that the ISO-11063 procedure yielded significantly less overall microbial DNA, (corroborated by measurement of the bacterial, archaeal and fungal densities by real-time PCR), whatever the soil is (Plassart *et al*., 2012). Furthermore, the analysis of fungal communities' structure with terminal restriction fragment length polymorphism (T-RFLP) patterns showed that the two non-ISO methods clearly outperformed the ISO-11063 method, leading to more significant variations because of soil type and management. Finally, one major conclusion of this study was that the non-ISO methods provided a better representativeness of soil DNA mainly due to use of the FastPrep®-24 bead-beating system, achieving lysis of the majority of cells with tough walls and particularly fungal cells, more efficiently than the usual bead beating (Ranjard *et al*., 2010; Rousk *et al*., 2010; Yarwood *et al*., 2010; Dequiedt *et al*., 2011; Plassart *et al*., 2012). Nevertheless, this comparative study was carried out using classical molecular approaches, i.e., quantitative PCR and community DNA fingerprinting through T-RFLP. Nowadays, high throughput sequencing technologies (e.g. 454 or Illumina) are readily available to assess microbial diversity with greater precision by obtaining hundreds of thousands of ribosomal rRNA gene sequences from a single metagenomic DNA (Roesch *et al*., 2007; Will *et al*., 2010; Maron *et al*., 2011). Nonetheless, the DNA extraction techniques previously described has never been evaluated with these new technologies, in terms of efficiency and representativeness, despite their widespread use in soil microbial diversity studies.

In the present study, the same three DNA extraction procedures, coupled with high throughput sequencing technology, were evaluated to identify a technique suitable to characterize the diversity and composition of bacterial and fungal communities simultaneously. The guideline standard ISO-11063, the custom GnS-GII and a custom DNA extraction procedure derived from the ISO-11063 standard (ISOm), were used to extract template DNA from five different soils with contrasting land-use and physico-chemical properties (Plassart *et al*., 2012). A meta-barcoded pyrosequencing technique, targeting the 16S and 18S rRNA genes, was used to characterize bacterial and fungal communities' richness [based on the number of operational taxonomic units (OTUs) and genera detected], diversity (using Shannon and Evenness indices) and composition (taxonomic affiliation of OTUs). We also measured the phylogenetic distance between sets of OTUs in a phylogenetic tree using the unifrac method to determine whether bacterial and fungal community compositions were influenced by the DNA extraction procedures.

## Results and discussion

Since the development of molecular tools to study soil microbial communities, it has been largely demonstrated that the characterization of these communities might be influenced by the method used to recover soil metagenomic DNA (Delmont *et al*., 2011b; Terrat *et al*., 2012). It is consequently essential to test the representativeness of soil DNA extraction methods in terms of bacterial and fungal organisms, which constitute a major part of the soil microbial community. Here, the efficacy of three soil DNA extraction methods (ISO-11063, ISOm and GnS-GII) was assessed on five soils with different physico-chemical characteristics and land use (Table 1) using a meta-barcoded pyrosequencing technique targeting bacterial and fungal communities. This approach was chosen because it is a recently developed powerful technique widely used for detailed phylogenetic and taxonomic surveys of microbial communities (Roesch *et al*., 2007; Rousk *et al*., 2010; Will *et al*., 2010; Lienhard *et al*., 2013a).

**Table 1:** Origin, physical and chemical parameters of the five French soils used

Soil	Collection site	Origin	Clay	Fine loam	Coarse loam	Fine sand	Coarse sand	Organic carbon	Total N	C/N	CaCO_3_	pH
C	Agricultural Site (Champdotre, Burgundy)	Crop soil	504	180	145	73	98	24.9	2.8	9	102	7.75
E	INRA Experimental Site (Epoisses, Burgundy)	Crop soil	392	320	228	34	26	16.5	1.65	10	2	7
F	Forest Observatory Plot (La Mailleraye-sur-Seine, Normandy)	Forest soil	101	167	205	217	310	103.3	3.1	34	< 1	3.8
L	INRA Experimental Site SOERE-ACBB (Lusignan, Poitou)	Grassland	175	369	304	73	79	13.2	1.33	9.92	< 1	6.6
R	INRA Experimental Site (Pierrelaye, Ile-de-France)	Crop soil	79	66	44	315	496	50.2	2.16	23.3	22	7.5

Clay, fine loam, coarse loam, fine sand and coarse sand, organic carbon, total N and calcium carbonate are given in mg g^−1^. Originally published and extracted from (Plassart *et al*., 2012).

### Influence of soil DNA extraction procedure on bacterial richness and diversity

Bacterial rRNA gene sequences were successfully amplified by PCR and sequenced from all soils using each of the three DNA extraction procedures (Table 2). After bioinformatic filters, 2322 high-quality reads per sample were kept, analyzed and taxonomically identified using a curated database derived from SILVA (Quast *et al*., 2013) (Table 3). Rarefaction curves of bacterial richness demonstrated that our sequencing depth allowed accurate description of the bacterial community diversity in each soil sample studied (Supporting Information Fig. S1).

**Table 2:** Bacterial richness and diversity indices of the five soils used

		Number of genera	OTUs (95% of similarity)	Shannon	Evenness
C	GnS	207.67 (± 5.73) *2*	485.67 (± 49.13) *1,2*	5.17 (± 0.13) *1,2*	0.84 (± 0.01) *1,2*
	ISO	207.33 (± 4.19) *1,2*	524.33 (± 15.11) *1,2*	5.15 (± 0.01) *1,2*	0.82 (± 0.00) *1,2*
	ISOm	205.50 (± 0.5) *1,2*	521.5 (± 2.5) *1,2*	5.24 (± 0.03) *1,2*	0.84 (± 0.01) *1,2*
E	GnS	205.67 (± 7.59) *2*	522.67 (± 50.37) *1,2*	5.16 (± 0.12) *1,2*	0.82 (± 0.01) *1,2*
	ISO	194.00 (± 5.89) *1,2*	545.33 (± 31.48) *1,2*	5.22 (± 0.06) *1,2*	0.83 (± 0.00) *1,2*
	ISOm	200.00 (± 7.48) *1,2*	498.67 (± 21.64) *1,2*	5.16 (± 0.02) *1,2*	0.80 (± 0.00) *1,2*
F	GnS	102.67 (± 11.14) *1*	329.33 (± 50.31) *1*	4.12 (± 0.26) *1*	0.71 (± 0.03) *1*
	ISO	111.00 (± 1.63) *1*	358.33 (± 31.56) *1*	4.33 (± 0.21) *1*	0.74 (± 0.03) *1*
	ISOm	97.33 (± 2.87) *1*	281.33 (± 12.5) *1*	3.99 (± 0.13) *1*	0.71 (± 0.02) *1*
L	GnS	234.33 (± 14.27) *1,2*	658.3 (± 22.48) *2*	5.58 (± 0.04) *2*	0.86 (± 0.00) *2*
	ISO	232.67 (± 2.87) *2*	668.67 (± 38.69) *2*	5.68 (± 0.06) *2*	0.87 (± 0.00) *2*
	ISOm	231.67 (± 11.09) *1,2*	692 (± 50.34) *2*	5.62 (± 0.09) *2*	0.86 (± 0.01) *2*
R	GnS	219.00 (± 9.80) *2*	561.67 (± 72.67) *1,2*	5.31 (± 0.15) *1,2*	0.84 (± 0.01) *1,2*
	ISO	223.33 (± 6.13) *2*	653.33 (± 39.35) *1,2*	5.63 (± 0.07) *1,2*	0.87 (± 0.00) *1,2*
	ISOm	231.00 (± 6.98) *2*	653.67 (± 33.89) *1,2*	5.5 (± 0.04) *1,2*	0.85 (± 0.00) *1,2*

The means were calculated with three replicates per soil (C, E, F, L and R) and procedure (ISO, ISOm and GnS-GII), and the standard errors of the means are indicated in parentheses. Significant differences between soils for the same procedure are indicated with numbers (1 – 1,2 – 2).

**Table 3:** Bioinformatic parameters and databases used in the analysis of bar-coded pyrosequencing results

Step	Parameter	Targeted rDNA Gene	
		16S	18S
Preprocessing	Length threshold	370	300
	Number of ambiguities tolerated	0	0
	Detection of proximal primer sequence	Complete and perfect	Complete and perfect
	Detection of distal primer sequence	No	Perfect, but potentially incomplete
Clustering	Chosen level of similarity (%)	95	95
	Ignoring differences in homopolymer lengths	Yes	Yes
Filtering	Chosen clustering similarity threshold	95	95
	Used taxonomic database	SILVA (r114)	SILVA (r111)
	Chosen taxonomic level	Phylum	Phylum
	Similarity or confidence threshold (%)	90	85
Homogenization	High-quality reads kept for each sample	2322	4378
Taxonomy	Used taxonomic database	SILVA (r114)	SILVA (r111)
	Method or tool of comparison	usearch	megablast
	Similarity or confidence threshold (%)	80	80
Analysis	Chosen level of similarity (%)	95	95
	Ignoring differences in homopolymer lengths	Yes	Yes
	Computation of a unifrac distance matrix	Yes	Yes

No significant differences were found between the three DNA extraction methods for the number of bacterial genera detected, the number of bacterial OTUs or for the Shannon and Evenness indices in any of the soils (Table 2). This means that neither the mechanical lysis step (using a mini bead-beater cell disruptor or the FastPrep®-24) nor the complete DNA extraction procedures had a significant effect on the evaluation of bacterial diversity parameters by meta-barcoding for the wide range of soil types and land uses tested (Table 1).

On the other hand, soil type did have an impact on bacterial richness and diversity indices, as significant differences were highlighted between soils, whatever the DNA extraction procedure (Table 2). Indeed, F and L soils (respectively the sandy acidic forest soil and the loamy grassland soil) were significantly different (*P* < 0.001) based on the number of OTUs, Shannon and Evenness indices. More precisely, the F soil had the lowest richness (number of OTUs and genera) and diversity, with for example a Shannon index of about 4.1 against 5.6 for the L soil (Table 2). This observation can be linked to particular physico-chemical characteristics, because the F soil had a pH of 3.8 and a C/N ratio of 34 (Dequiedt *et al*., 2011; Lienhard *et al*., 2013b). Several studies have highlighted that bacterial richness had a positive correlation with soil pH (Fierer and Jackson, 2006; Lauber *et al*., 2008; Terrat *et al*., 2012) and a negative correlation with C/N ratio (Kuramae *et al*., 2012). Indeed, a high C/N ratio is generally typical of a large recalcitrant organic matter content that is unfavourable for bacterial growth (Boer *et al*., 2005). However, the sandy crop soil R, also harbouring a C/N ratio of the same magnitude (23.3), holds a greater richness of OTUs and genera than the forest soil (Table 2). This might partly be due to either the high sand content (Table 1), which increases soil microscale heterogeneity and stimulates the bacterial richness (Chau *et al*., 2011) or an alkaline pH (7.5) favouring bacterial richness (Fierer and Jackson, 2006; Lauber *et al*., 2008; Terrat *et al*., 2012).

Altogether, our results confirmed that bacterial diversity and richness can be strongly linked to soil characteristics and especially soil pH, organic matter and texture (Fierer and Jackson, 2006; Lauber *et al*., 2008; Kuramae *et al*., 2012; Terrat *et al*., 2012; Lienhard *et al*., 2013b). All DNA extraction procedures tested gave enough and similar sensitivity to detect changes between indigenous bacterial communities of soils differing by their characteristics and management. These data also support the idea that a study limited to these diversity indices could not be sufficient to determine whether a DNA extraction procedure is more powerful than another to describe soil bacterial communities and that it might be completed by a more detailed bacterial community composition analysis.

### Influence of soil DNA extraction procedure on fungal richness and diversity

Using the same DNA extracts as for the bacterial analysis (three DNA extraction procedures applied to five soils), 18S rRNA gene sequences were successfully amplified and sequenced from all samples (Table 4). Homogenized high-quality reads (4378 per sample) were then analyzed using taxonomically dependent and independent analyses to determine fungal richness and diversity (Table 3). As for bacteria, the rarefaction curves of fungal richness confirmed that the number of high-quality reads allowed accurate description of the fungal community diversity in each soil sample studied (Supporting Information Fig. S1).

**Table 4:** Fungal richness and diversity indices of the five French soils used

		Number of *genera*	OTUs (95% of similarity)	Shannon	Evenness
C	GnS	116.00 (± 11.43) *1*	350.33 (± 42.32) *1,2*	3.72 (± 0.05) *1,2*	0.64 (± 0.01) *1,2*
	ISO	92.33 (± 15.69) *1*	273.67 (± 63.67) *1*	3.31 (± 0.33) *1*	0.59 (± 0.04) *1,2*
	ISOm	118.67 (± 9.67) *1*	340.67 (± 68.23) *1,2*	3.73 (± 0.15) *1,2*	0.64 (± 0.01) *1,2*
E	GnS	128.00 (± 13.74) *1*	287.33 (± 30.58) *1*	3.39 (± 0.08) *1,2*	0.6 (± 0.01) *1,2*
	ISO	108.33 (± 9.81) *1*	239.33 (± 31.54) *1*	3.34 (± 0.22) *1*	0.61 (± 0.03) *1,2*
	ISOm	125.67 (± 6.55) *1*	289 (± 33.66) *1*	3.54 (± 0.18) *1,2*	0.63 (± 0.02) *1,2*
F	GnS	129.67 (± 8.34) *1*	249.67 (± 11.15) *1*	2.98 (± 0.05) *1*	0.54 (± 0.01) *1*
	ISO	136.00 (± 7.79) *2*	312 (± 35.36) *1*	3.19 (± 0.12) *1*	0.56 (± 0.01) *1*
	ISOm	140.33 (± 10.14) *1*	267 (± 24.91) *1*	3.27 (± 0.18) *1*	0.59 (± 0.03) *1*
L	GnS	127.33 (± 9.29) *a.1*	416.33 (± 89.46) *2*	4.05 (± 0.21) *2*	0.67 (± 0.01) *2*
	ISO	89.67 (± 5.79) *b.1*	353.33 (± 51.45) *1*	3.89 (± 0.09) *1*	0.66 (± 0.03) *1,2*
	ISOm	129.00 (± 6.48) *a.1*	382.33 (± 71.82) *2*	3.74 (± 0.49) *2*	0.63 (± 0.06) *2*
R	GnS	141.33 (± 13.82) *1*	407.33 (± 84.94) *2*	3.9 (± 0.2) *1,2*	0.65 (± 0.01) *1,2*
	ISO	111.00 (± 11.00) *1,2*	399.00 (± 30.00) *2*	4.14 (± 0.01) *1*	0.69 (± 0.01) *2*
	ISOm	135.00 (± 12.68) *1*	407 (± 67.38) *2*	3.94 (± 0.12) *1,2*	0.66 (± 0.01) *1,2*

The means were calculated with three replicates per soil (C, E, F, L and R) and procedure (ISO, ISOm and GnS-GII), and the standard errors of the means are indicated in parentheses. Significant differences between procedures for the same soil are indicated by letters (*a, b*), and significant differences between soils for the same procedure are indicated with numbers (1 – 1,2 – 2).

With regard to the number of detected genera, the numbers of OTUs and the computed indices (Shannon and Evenness), significant differences among the three DNA extraction procedures were recorded only for the L soil (Table 4), in which a lower number of fungal genera was significantly detected using the ISO procedure (*P* = 0.003). Moreover, in all the other soils but F, the number of genera recovered followed the same trend, with lower values detected for the ISO, than what was observed for the two other procedures. These genera missed by the ISO demonstrate that fungal diversity can be skewed using this procedure. As the main difference among the ISO and the two other procedures is the soil-grinding step; we can hypothesize that the traditional bead-beating system is not sufficient to lyze some fungal cells. Indeed, many fungi have cell walls that impede lysis and the recovery of nucleic acids (Fredricks *et al*., 2005). The mechanical lysis step of the ISOm and GnS-GII procedures was strongly optimized in terms of type and size of the glass beads as well as in terms of the strength and duration of grinding using the FastPrep®-24 (Terrat *et al*., 2012).

When fungal diversity was compared between soils, significant differences in the Shannon and Evenness indices (*P* < 0.05) and in the number of OTUs (*P* < 0.1) were observed whatever the DNA extraction procedure (Table 4). More precisely, the acidic forest soil F harboured the lowest richness and diversity, and the alkaline sandy crop soil R the highest (Table 4). These differences could be explained by several soil physico-chemical parameters, namely their contrasting pH (3.8 against 7.75), but also their C/N ratio (34 against 23.3) (Table 1). Although extreme environments like acidic soils may provide suitable biotopes for fungi (Baker and Banfield, 2003; Butinar *et al*., 2005), the lowest richness and diversity was detected in the acidic forest soil F, indicating that other physico-chemical parameters can limit fungal communities. Thus, a high C/N ratio is typical of soil systems with a low rate of organic matter degradation because of the presence of a high proportion of recalcitrant organic matter (Kuramae *et al*., 2012). Strickland and Rousk (2010) demonstrated in a previous study that the optimal C/N for fungi is expected to range from 5 to 15; i.e. closer to the C/N of the sandy crop soil R than to the ratio of the forest soil F, which has a higher carbon content. Focusing on the number of fungal genera recovered by the three DNA extraction procedures, only the ISO allowed the detection of significant differences between soils. This finding has to be seriously questioned because we demonstrated in the previous paragraph that the ISO underestimates the number of fungal genera.

### Influence of soil DNA extraction procedure on bacterial community composition

**Fig 1:**
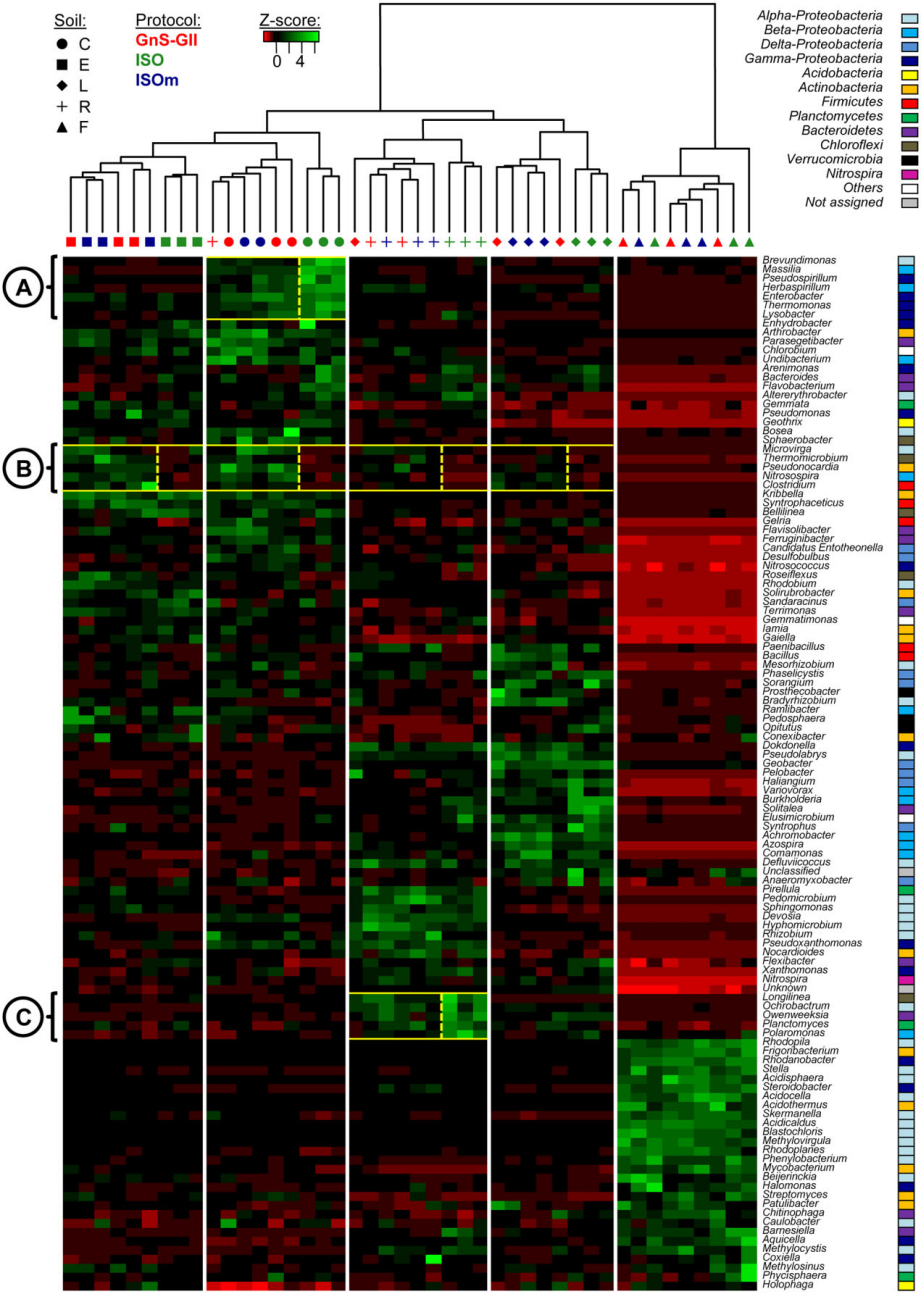
Heat map comparison of the dominant bacterial genera detected in soils according to extraction procedures. The five different soils (C, E, F, L, R) were organized based on the UPGMA dendrogram of unifrac distances (weighted and normalized) between soil samples according to the three DNA extraction procedures (ISO-11063, GnS-GII and ISOm). The legend shows the *Z*-scores (relative abundances are expressed as median centred *Z*-scores between all samples, and the colours scaled to standard deviations). Subcells A, B and C in the heat map have been highlighted by yellow squares and numbered to identify significant differences in the relative abundance of particular bacterial genera according to DNA extraction procedure.

The bacterial community composition in the five soils was compared by computing the unifrac distances on a phylogenetic tree (Lozupone and Knight, 2005). In addition to analyzing the phylogenetic distances, we also compared the bacterial communities' compositions based on the relative abundance of the bacterial genera detected in the samples (Fig. 1). Due to the size and variability of the genus table, only the most highly represented bacterial genera in the samples (i.e. only those for which the sum of the relative abundances of the genus in all samples was higher than 5%) were identified and mapped.

The clustering of soil bacterial communities indicated that replicates from the same soil were more similar to each other than replicates from other soils, whatever the DNA extraction procedure (Fig. 1). This observation demonstrated the good reproducibility between replicates for each type of soil DNA extraction procedure even if, surprisingly, two GnS-GII replicates from soils L and R seemed to be erroneously clustered. More precisely, two main clusters were identified, sorting the samples from the acidic forest soil F (which hosted a very different bacterial composition) apart from the four other soils (Fig. 1). Four sub-clusters could also be defined, each one grouping samples from each of the four soils and confirming that the studied soils hosted distinct bacterial communities, as already demonstrated by DNA fingerprinting approach (Plassart *et al*., 2012). This observation demonstrated the good reproducibility between replicates for ISO and ISOm procedures. However, even if clustering revealed that soil type had a more important effect on bacterial composition than the DNA extraction procedure, it is interesting to note that this latter could induce significant variations (Fig. 1). For all soils (except the forest soil F), the bacterial diversity profiles resulting from the ISOm and GnS-GII DNA extraction procedures grouped together (i.e. were not discriminated by the unifrac analysis), but were different from those obtained with the ISO-11063 procedure (Fig. 1). These observations confirm the influence of soil DNA extraction procedure on soil bacterial composition and especially the clear distinction between ISO-11063 and the two other procedures, potentially explained by differences in the soil-grinding methods (as discussed above for fungal richness and diversity). These differences were also confirmed by a more detailed analysis of bacterial composition (Fig. 1, subcells A–C). For example, the genus *Brevundimonas* was more detected (*P* < 0.05) with the ISO-11063 procedure than with the two others in the clayey crop soil C (Fig. 1, subcell A), as were the genera *Massilia*, *Pseudospirillum*, *Herbaspirillum*, *Enterobacter*, *Thermomonas* and *Lysobacter*. Similarly, the genus *Polaromonas* was more detected in the sandy crop soil R (Fig. 1, subcell C), but not in the other soils. On the contrary, the genera *Clostridium*, *Nitrosospira*, *Microvirga* and *Pseudonocardia* were respectively less detected (*P* < 0.05) with ISO-11063 than with the ISOm and GnS-GII procedures in soils C, E, L and R (Fig. 1, subcell B). Because the genera *Clostridium* or *Pseudonocardia* are known to be potentially recalcitrant to mechanical lysis, because of their spore-forming ability (Kaewkla and Franco, 2011; Yang and Ponce, 2011), their lower detection with the ISO-11063 procedure may be explained by the less efficient mechanical lysis (bead beating) of this procedure, compared with the two others, which are based on FastPrep®-24 grinding (Plassart *et al*., 2012; Terrat *et al*., 2012).

### Influence of soil DNA extraction procedure on fungal community composition

**Fig 2:**
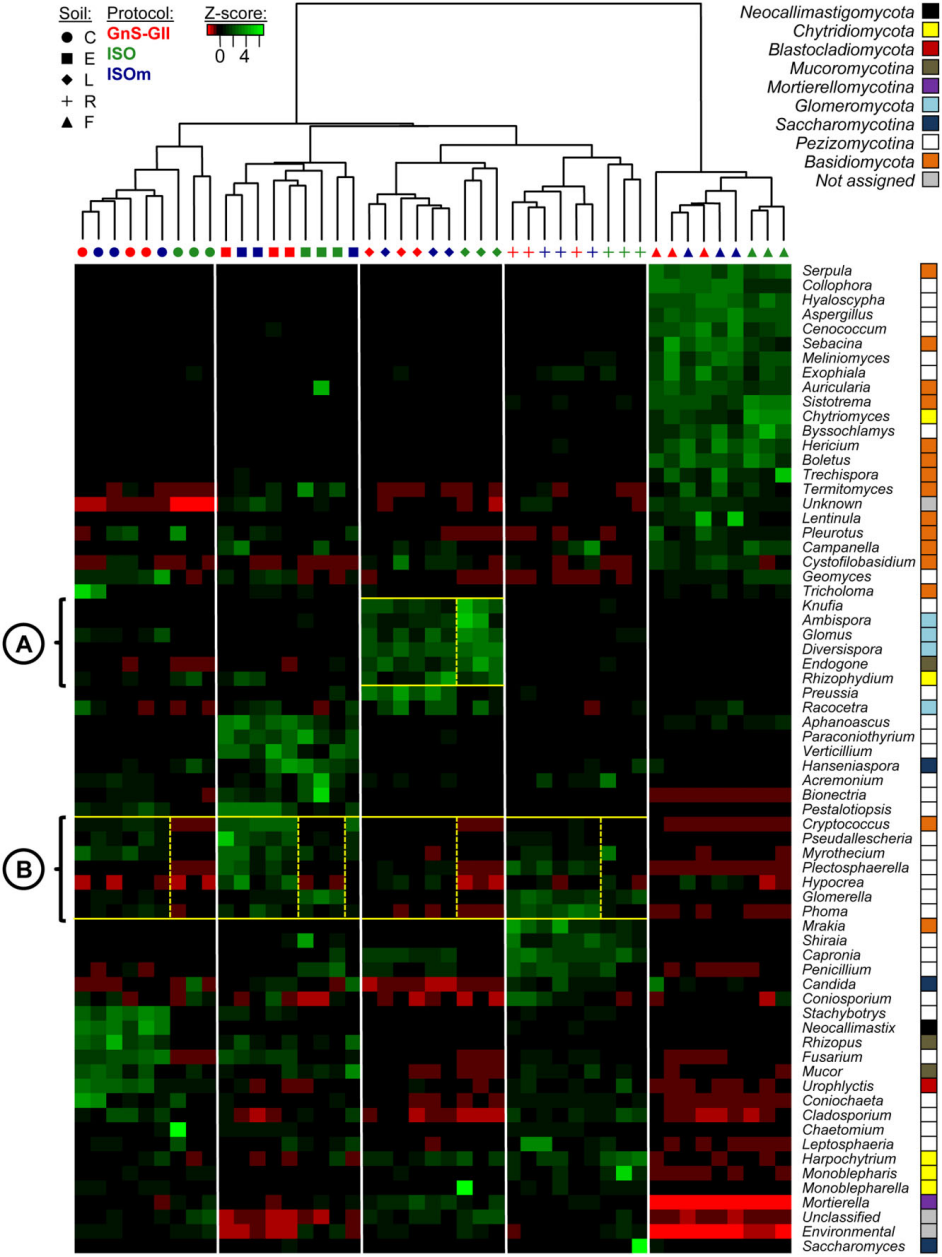
Heat map comparison of the dominant fungal genera detected in soils according to extraction procedures. The five different soils (C, E, F, L, R) were organized based on the UPGMA dendrogram of unifrac distances (weighted and normalized) between soil samples according to the three DNA extraction procedures (ISO-11063, GnS-GII and ISOm). The legend shows the *Z*-scores (relative abundances are expressed as median centred *Z*-scores between all samples, and the colours scaled to standard deviations). Subcells A and B in the heat map have been highlighted by yellow squares and numbered to identify significant differences in the relative abundance of particular fungal genera according to DNA extraction procedure.

As with the bacterial communities, the fungal communities' composition in all soils was compared by using the unifrac distances and determining the most highly represented fungal genera in the samples (Fig.  2). The unifrac dendrogram revealed a better discrimination of fungal composition between soils than between DNA extraction methods, demonstrating a good reproducibility between replicates for all procedures (Fig. 2). Moreover, as for the bacterial communities, the same clustering organization was obtained for fungal communities, revealing a significant distinction between the forest soil and the other soils. This observation corroborates other studies in which soil characteristics (e.g. pH, texture, C/N) were shown to impact fungal community diversity and composition (Rousk *et al*., 2010; Strickland and Rousk, 2010; McGuire *et al*., 2013). The fungal populations in this acidic soil clearly differed from those of the other soils, with a dominance of the *Basidiomycota phylum* (e.g. genera *Sebacina*, *Boletus*, *Pleurotus or Hericium*), which is common in forest soils (Buée *et al*., 2009).

For soils F, L and R, the patterns of the fungal communities resulting from the ISO-11063 procedure were discriminated from those obtained with the two non-ISO protocols (Fig. 2). This observation evidenced that in these soils, the fungal community compositions detected with the ISO-11063 differed from those detected with the non-ISO procedures. More precisely, in the loamy grassland soil L, several *genera* (e.g. *Knufia* and *Diversispora*) were more detected (*P* < 0.1) with the ISO-11063 protocol (Fig. 2, subcell A). However, this positive impact of the ISO-11063 procedure was only visible for this particular soil. On the contrary, the genera *Myrothecium*, *Cryptococcus*, *Glomerella* and *Plectosphaerella* were respectively less detected (*P* < 0.05) with the ISO-11063 protocol than with the other methods in soils C, E, L and R (Fig. 2, subcell B), as *Pseudallescheria* in soils C, E and R, and *Hypocrea* in soils E and L (*P* < 0.1). This would be of great importance in ecological studies as some of these genera (e.g. *Cryptococcus*, *Pseudallescheria*, *Hypocrea* and *Plectosphaerella*) are saprotrophic fungi known to play key roles in organic matter turnover (Martínez *et al*., 2003; Jaklitsch *et al*., 2005; Buée *et al*., 2009; McGuire *et al*., 2013). Moreover, the genus *Pseudallescheria*, which has been found in compost-amended or heavily hydrocarbon-polluted soils, can be used as an indicator of soil disturbance (April *et al*., 1998). Therefore, the ISO-induced underrepresentation of these genera could lead to a misinterpretation of the functioning of an ecosystem.

This difference in community composition is, together with the lower number of fungal genera recovered with the ISO described earlier, a clue indicating that the ISO procedure may not be the most appropriate to investigate soil fungal communities. Besides, these differences are thought to be due to the less efficient mechanical lysis of soil with the ISO-11063 procedure; the classical system seems not to break open as many cells as the FastPrep®-24 bead-beating system, particularly in the case of fungal cells with tough walls. This is why the ISOm and GnS-GII methods are thought to be more efficient at extracting fungal DNA from different types of soils. This conclusion strengthens the idea that the physical lysis step is of crucial importance in a soil DNA extraction procedure (Feinstein *et al*., 2009; İnceoğlu *et al*., 2010; Delmont *et al*., 2011b). This finding is in agreement with previous comparisons of these DNA extraction procedures based on quantitative PCR and community DNA fingerprinting (Plassart *et al*., 2012).

## Conclusion

In the context of modern microbial ecology, where investigations to describe the whole soil microbiota in numerous samples are carried out on a very large scale, the importance of using a single, standardized soil DNA extraction procedure is paramount. Among the three DNA extraction procedures evaluated in this study, the GnS-GII introduced some heterogeneity in bacterial composition between replicates, and the ISO-11063 DNA caused an underrepresentation of several fungal groups of ecological interest. Therefore, the ISOm procedure provides a better snapshot of bacterial and fungal communities.

## Experimental procedures

### Soil samples

Five soils were chosen for their contrasting land-use and physico-chemical characteristics (Table 1) (Plassart *et al*., 2012). All necessary permits were obtained from the respective land owners (INRA, ADEME and private owners). For each soil, three independent replicates were collected at a depth of 20 cm [fully described in (Plassart *et al*., 2012)]. Physico-chemical characteristics (pH, texture, organic carbon, total N and CaCO_3_) were analyzed, using international standard procedures, by the Soil Analysis Laboratory at INRA (Arras, France, http://www.lille.inra.fr/las).

### Soil DNA extraction, purification and quantification

Three different procedures were tested: the GnS-GII protocol, the ISO-11063 standard and the ISOm. All three procedures are adapted to extract DNA from 1 g of soil (dry weight) and have already been described by Plassart and colleagues (2012).

#### ISO-11063 procedure

This protocol is a version of the ISO-11063 standard (Martin-Laurent *et al*., 2001; Petric *et al*., 2011). Soil was added to a bead-beating tube containing 2 g of glass beads of 106 μm diameter and eight glass beads of 2 mm diameter. Each soil sample was mixed with a solution of 100 mM Tris-HCl (pH 8), 100 mM EDTA (pH 8), 100 mM NaCl, 2% (w/v) polyvinylpyrrolidone (40 g mol^−1^) and 2% (w/v) sodium dodecyl sulfate. The tubes were then shaken for 30 s at 1600 r.p.m. in a mini bead-beater cell disruptor (Mikro-Dismembrator, Braun Biotech International), then incubated for 10 min at 70°C and centrifuged at 14,000*g* for 1 min. After removing the supernatant, proteins were precipitated, with 1/10 volume of 3 M sodium acetate prior to centrifugation (14,000*g* for 5 min at 4°C). Finally, nucleic acids were precipitated by adding 1 volume of ice-cold isopropanol. The DNA pellets obtained after centrifugation (14,000*g* for 5 min at 4°C) were washed with 70% ethanol (full details are described in (Martin-Laurent *et al*., 2001; Philippot *et al*., 2010; Petric *et al*., 2011).

#### ISOm procedure

This protocol is a modified version of ISO-11063 standard as it includes a different mechanical lysis step (FastPrep® bead-beating instead of the recommended bead beating). Soil was added to 15 ml of Falcon tube containing 2.5 g of 1.4 mm diameter ceramic beads, 2 g of 106 μm diameter silica beads and four glass beads of 4 mm diameter. Each soil sample was mixed with a solution of 100 mM Tris-HCl (pH 8), 100 mM EDTA (pH 8), 100 mM NaCl, 2% (w/v) polyvinylpyrrolidone (40 g mol^−1^) and 2% (w/v) sodium dodecyl sulfate. The tubes were then shaken for 3 × 30 s at 4 m sec^−1^ in a FastPrep®-24 (MP-Biomedicals, NY, USA), before incubation for 10 min at 70°C and centrifugation at 14,000*g* for 1 min. After removing the supernatant, proteins were precipitated with 1/10 volume of 3 M sodium acetate prior to centrifugation (14,000*g* for 5 min at 4°C). Finally, nucleic acids were precipitated by adding 1 volume of ice-cold isopropanol. The DNA pellets obtained after centrifugation (14,000*g* for 5 min at 4°C) were washed with 70% ethanol.

#### GnS-GII procedure

This DNA extraction procedure was initially developed and optimized by the GenoSol platform (Terrat *et al*., 2012). Soil was added to 15 ml of Falcon tube containing 2.5 g of 1.4 mm diameter ceramic beads, 2 g of 106 μm diameter silica beads and four glass beads of 4 mm diameter. Each soil sample was mixed with a solution of 100 mM Tris-HCl (pH 8), 100 mM EDTA (pH 8), 100 mM NaCl, 2% (w/v) and 2% (w/v) sodium dodecyl sulfate. The tubes were then shaken for 3 × 30 s at 4 m sec^−1^ in a FastPrep®-24 (MP-Biomedicals, NY, USA), before incubation for 30 min at 70°C and centrifugation at 7,000*g* for 5 min at 20°C. After removing the supernatant, proteins were precipitated with 1/10 volume of 3 M sodium acetate prior to centrifugation (14,000*g* for 5 min at 4°C). Finally, nucleic acids were precipitated by adding 1 volume of ice-cold isopropanol. The DNA pellets obtained after centrifugation (14,000*g* for 5 min at 4°C) were washed with 70% ethanol.

#### Purification and quantification procedure

As the DNA purification step is not part of the evaluated protocols to avoid additional biases among the three procedures and only compare the extraction step, all crude soil DNA extracts were purified and quantified using the same procedure (Ranjard *et al*., 2003; Plassart *et al*., 2012). Briefly, 100 μl aliquots of crude DNA extracts were loaded onto PVPP (polyvinylpolypyrrolidone) Microbiospin minicolumns (Bio-Rad) and centrifuged for 4 min at 1000*g* and 10°C. Eluates were then collected and purified for residual impurities using the Geneclean Turbo kit (MP-Biomedicals, NY, USA). Purified DNA extracts were quantified using the PicoGreen staining Kit (Molecular Probes, Paris, France).

### Pyrosequencing of 16S and 18S rRNA gene sequences

Microbial diversity was determined for each biological replicate and for each soil (C, F, E, L and R) by 454 pyrosequencing of ribosomal genes. A 16S rRNA gene fragment with sequence variability and appropriate size (about 450 bases) for 454 pyrosequencing was amplified using the primers F479 (5′-CAGCMGCYGCNGTAANAC-3′) and R888 (5′-CCGYCAATTCMTTTRAGT-3′) (Supporting Information Table S1 for *in silico* match analysis, Terrat *et al*. 2014). For each sample, 5 ng of DNA were used for a 25 μl of PCR conducted under the following conditions: 94°C for 2 min, 35 cycles of 30 s at 94°C, 52°C for 30 s and 72°C for 1 min, followed by 7 min at 72°C. The PCR products were purified using a MinElute gel extraction kit (Qiagen, Courtaboeuf, France) and quantified using the PicoGreen staining Kit (Molecular Probes, Paris, France). Similarly, an 18S rRNA gene fragment of about 350 bases was amplified using the primers FR1 (5′-ANCCATTCAATCGGTANT-3′) and FF390 (5′-CGATAACGAACGAGACCT-3′) (Prevost-Boure *et al*., 2011) under the following PCR conditions: 94°C for 3 min, 35 cycles of 1 min at 94°C, 52°C for 1 min and 72°C for 1 min, followed by 5 min at 72°C. A second PCR of nine cycles was then conducted twice for each sample under similar PCR conditions with purified PCR products and 10 base pair multiplex identifiers added to the primers at 5′ position to specifically identify each sample and avoid PCR bias. Finally, the duplicate PCR products were pooled, purified and quantified as previously described. Pyrosequencing was then carried out on a GS FLX Titanium (Roche 454 Sequencing System) by Genoscreen (Lille, France).

### Bioinformatic analysis of 16S and 18S rRNA gene sequences

Bioinformatic analyses were done using the GnS-PIPE initially developed by the Genosol platform (INRA, Dijon, France) (Terrat *et al*., 2012) and recently optimized. The parameters chosen for each bioinformatic step can be found in Table 3. First, all the 16S and 18S raw reads were sorted according to the multiplex identifier sequences. The raw reads were then filtered and deleted based on (i) their length, (ii) their number of ambiguities (Ns) and (iii) their primer(s) sequence(s). A perl program was then applied for rigorous dereplication (i.e. clustering of strictly identical sequences). The dereplicated reads were then aligned using infernalalignment (Cole *et al*., 2009), and clustered into OTU using a perl program that groups rare reads to abundant ones, and does not count differences in homopolymer lengths. A filtering step was then carried out to check all single singletons (reads detected only once and not clustered, which might be artefacts, such as PCR chimeras) based on the quality of their taxonomic assignments. Finally, in order to compare the data sets efficiently and avoid biased community comparisons, the reads retained were homogenized by random selection closed to the lowest dataset.

The retained high-quality reads were used for (i) taxonomy-independent analyses, determining several diversity and richness indices using the defined OTU composition at the genus level and (ii) taxonomy-based analysis using similarity approaches against dedicated reference databases from SILVA (Quast *et al*., 2013) (see Table 3). The raw data sets are available on the European Bioinformatics Institute database system under project accession number PRJEB4825.

### Statistical analyses

The effects of the DNA extraction procedure on bacterial and fungal diversities were tested by analysis of variance (multiple paired comparisons). The effects of the DNA extraction procedure on bacterial and fungal community compositions were assessed by Kruskal–Wallis tests. All statistical analyses were performed under xlstat software (Addinsoft®). The bacterial and fungal communities from all samples were also compared by using unifrac (Lozupone and Knight, 2005), based on the 16S and 18S phylogenetic trees computed with fasttree(Price *et al*., 2010).

## Supporting Information

**Fig. S1.** Rarefaction curves of bacterial and fungal OTUs detected in soils according to extraction procedures.

**Table S1.** Detailed hit frequencies (%) of the in silico analysis of the F479/R888 primer set for Bacteria, Archaea and Eukaryota.
