# Supplementary material for: Meta-barcoded evaluation of the ISO standard 11063 DNA extraction procedure to characterize soil bacterial and fungal community diversity and composition
Source: Microb Biotechnol. 2014 Sep 4;8(1):131–42. doi: 10.1111/1751-7915.12162 (PMC4321379; doi:10.1111/1751-7915.12162)
Supplement: Supplementary file 1 — Fig. S1. Rarefaction curves of bacterial and fungal OTUs detected in soils according to extraction procedures. [file mbt20008-0131-sd1.docx]

**Supporting Information S1. Rarefaction curves of bacterial and fungal OTUs detected in soils according to extraction procedures.** Each plot represents the three replicates of each DNA extraction procedure (ISO-11063 in green, GnS-GII in red and ISOm in blue) for a given soil (C, E, F, L or R).
