# Supplementary material for: Meta-barcoded evaluation of the ISO standard 11063 DNA extraction procedure to characterize soil bacterial and fungal community diversity and composition
Source: Microb Biotechnol. 2014 Sep 4;8(1):131–42. doi: 10.1111/1751-7915.12162 (PMC4321379; doi:10.1111/1751-7915.12162)
Supplement: Supplementary file 2 — Table S1. Detailed hit frequencies (%) of the in silico analysis of the F479/R888 primer set for Bacteria, Archaea and Eukaryota. [file mbt20008-0131-sd2.docx]

**Supporting Information S2. Detailed hit frequencies (%) of the *in silico* analysis of the F479/R888 primer set for Bacteria, Archaea, and Eukaryota.** The analysis allowed k mismatches, k ranging from 0 (original primer set sequences) to 1 (test of primer set sequences with one tolerated mismatch). The sequences investigated were SILVA, and direct extraction of every SSU rRNA sequence from EMBL using acnuc, and also a dedicated reference database of 18S eukaryotic sequences which have been thoroughly analyzed and annotated.

|  |  |  |  | **Matched sequences** | | **Hit frequencies (%)** | |
| --- | --- | --- | --- | --- | --- | --- | --- |
| **Origin** | ***Phylum*** | ***Class*** | **Total number of sequences** | **k = 0** | **k = 1** | **k = 0** | **k = 1** |
| *Chloroplast* | *Cyanobacteria* | *Chloroplast* | 4389 | 4036 | 4248 | 92,0 | 96,8 |
| *Mitochondria* | *Proteobacteria* | *Alphaproteobacteria* | 567 | 347 | 389 | 61,2 | 68,6 |
|  |  |  |  |  |  |  |  |
| *Archaea* | *Crenarchaeota* | *AK31* | 31 | 30 | 30 | 96,8 | 96,8 |
| *Archaea* | *Crenarchaeota* | *AK56* | 10 | 9 | 9 | 90,0 | 90,0 |
| *Archaea* | *Crenarchaeota* | *AK59* | 63 | 56 | 58 | 88,9 | 92,1 |
| *Archaea* | *Crenarchaeota* | *AK8* | 11 | 8 | 9 | 72,7 | 81,8 |
| *Archaea* | *Crenarchaeota* | *D-F10* | 15 | 12 | 13 | 80,0 | 86,7 |
| *Archaea* | *Crenarchaeota* | *FS243A-60* | 10 | 9 | 10 | 90,0 | 100,0 |
| *Archaea* | *Crenarchaeota* | *OPPD003* | 7 | 7 | 7 | 100,0 | 100,0 |
| *Archaea* | *Crenarchaeota* | *Papm3A43* | 2 | 2 | 2 | 100,0 | 100,0 |
| *Archaea* | *Crenarchaeota* | *TOTO-A6-15* | 3 | 3 | 3 | 100,0 | 100,0 |
| *Archaea* | *Crenarchaeota* | *Thermoprotei* | 1045 | 936 | 1007 | 89,6 | 96,4 |
| *Archaea* | *Crenarchaeota* | *Z273FA48* | 2 | 1 | 2 | 50,0 | 100,0 |
| *Archaea* | *Crenarchaeota* | *pMC2A209* | 5 | 2 | 2 | 40,0 | 40,0 |
| *Archaea* | *Crenarchaeota* | *pSL12* | 13 | 13 | 13 | 100,0 | 100,0 |
| *Archaea* | *Euryarchaeota* | *Archaeoglobi* | 115 | 104 | 112 | 90,4 | 97,4 |
| *Archaea* | *Euryarchaeota* | *Halobacteria* | 3742 | 2865 | 3475 | 76,6 | 92,9 |
| *Archaea* | *Euryarchaeota* | *Methanobacteria* | 1557 | 1439 | 1503 | 92,4 | 96,5 |
| *Archaea* | *Euryarchaeota* | *Methanococci* | 141 | 124 | 133 | 87,9 | 94,3 |
| *Archaea* | *Euryarchaeota* | *Methanomicrobia* | 5358 | 4898 | 5148 | 91,4 | 96,1 |
| *Archaea* | *Euryarchaeota* | *Methanopyri* | 13 | 0 | 13 | 0,0 | 100,0 |
| *Archaea* | *Euryarchaeota* | *Thermococci* | 386 | 334 | 346 | 86,5 | 89,6 |
| *Archaea* | *Euryarchaeota* | *Thermoplasmata* | 4068 | 3238 | 3809 | 79,6 | 93,6 |
| *Archaea* | *Euryarchaeota* | *pCIRA-13* | 3 | 2 | 3 | 66,7 | 100,0 |
|  |  |  |  |  |  |  |  |
| *Bacteria* | *Acidobacteria* | *Acidobacteria* | 8048 | 7604 | 7907 | 94,5 | 98,2 |
| *Bacteria* | *Acidobacteria* | *Holophagae* | 953 | 899 | 935 | 94,3 | 98,1 |
| *Bacteria* | *Acidobacteria* | *RB25* | 279 | 267 | 277 | 95,7 | 99,3 |
| *Bacteria* | *Actinobacteria* | *Acidimicrobiia* | 3783 | 1902 | 3705 | 50,3 | 97,9 |
| *Bacteria* | *Actinobacteria* | *Actinobacteria* | 33891 | 29468 | 33349 | 86,9 | 98,4 |
| *Bacteria* | *Actinobacteria* | *Coriobacteria* | 1759 | 1613 | 1712 | 91,7 | 97,3 |
| *Bacteria* | *Actinobacteria* | *FFCH16263* | 2 | 2 | 2 | 100,0 | 100,0 |
| *Bacteria* | *Actinobacteria* | *KIST-JJY010* | 3 | 3 | 3 | 100,0 | 100,0 |
| *Bacteria* | *Actinobacteria* | *MB-A2-108* | 199 | 185 | 192 | 93,0 | 96,5 |
| *Bacteria* | *Actinobacteria* | *Nitriliruptoria* | 91 | 88 | 90 | 96,7 | 98,9 |
| *Bacteria* | *Actinobacteria* | *OPB41* | 90 | 82 | 86 | 91,1 | 95,6 |
| *Bacteria* | *Actinobacteria* | *Rubrobacteria* | 234 | 35 | 217 | 15,0 | 92,7 |
| *Bacteria* | *Actinobacteria* | *TakashiAC-B11* | 16 | 16 | 16 | 100,0 | 100,0 |
| *Bacteria* | *Actinobacteria* | *Thermoleophilia* | 1113 | 1049 | 1093 | 94,2 | 98,2 |
| *Bacteria* | *Aquificae* | *Aquificae* | 890 | 793 | 870 | 89,1 | 97,8 |
| *Bacteria* | *Armatimonadetes* | *Armatimonadia* | 144 | 122 | 141 | 84,7 | 97,9 |
| *Bacteria* | *Bacteroidetes* | *AMV16* | 16 | 16 | 16 | 100,0 | 100,0 |
| *Bacteria* | *Bacteroidetes* | *BD2-2* | 214 | 193 | 204 | 90,2 | 95,3 |
| *Bacteria* | *Bacteroidetes* | *BSV13* | 49 | 48 | 49 | 98,0 | 100,0 |
| *Bacteria* | *Bacteroidetes* | *Bacteroidia* | 45142 | 42677 | 44306 | 94,5 | 98,1 |
| *Bacteria* | *Bacteroidetes* | *Cytophagia* | 3610 | 3204 | 3550 | 88,8 | 98,3 |
| *Bacteria* | *Bacteroidetes* | *DUNssu192* | 4 | 4 | 4 | 100,0 | 100,0 |
| *Bacteria* | *Bacteroidetes* | *Flavobacteria* | 9328 | 8801 | 9109 | 94,4 | 97,7 |
| *Bacteria* | *Bacteroidetes* | *Ika33* | 8 | 8 | 8 | 100,0 | 100,0 |
| *Bacteria* | *Bacteroidetes* | *ML602M-17* | 11 | 11 | 11 | 100,0 | 100,0 |
| *Bacteria* | *Bacteroidetes* | *SB-1* | 186 | 172 | 182 | 92,5 | 97,8 |
| *Bacteria* | *Bacteroidetes* | *SB-5* | 92 | 87 | 90 | 94,6 | 97,8 |
| *Bacteria* | *Bacteroidetes* | *SM1A07* | 16 | 13 | 16 | 81,3 | 100,0 |
| *Bacteria* | *Bacteroidetes* | *Sphingobacteria* | 5252 | 4926 | 5131 | 93,8 | 97,7 |
| *Bacteria* | *Bacteroidetes* | *WCHB1-32* | 65 | 63 | 64 | 96,9 | 98,5 |
| *Bacteria* | *Bacteroidetes* | *vadinHA17* | 442 | 406 | 429 | 91,9 | 97,1 |
| *Bacteria* | *Caldiserica* | *Caldisericia* | 98 | 5 | 96 | 5,1 | 98,0 |
| *Bacteria* | *Chlamydiae* | *Chlamydiae* | 359 | 4 | 352 | 1,1 | 98,1 |
| *Bacteria* | *Chlorobi* | *Chlorobia* | 632 | 286 | 619 | 45,3 | 97,9 |
| *Bacteria* | *Chlorobi* | *Ignavibacteria* | 317 | 300 | 310 | 94,6 | 97,8 |
| *Bacteria* | *Chloroflexi* | *Anaerolineae* | 5618 | 1940 | 4730 | 34,5 | 84,2 |
| *Bacteria* | *Chloroflexi* | *Caldilineae* | 836 | 661 | 810 | 79,1 | 96,9 |
| *Bacteria* | *Chloroflexi* | *Chloroflexi* | 351 | 319 | 338 | 90,9 | 96,3 |
| *Bacteria* | *Chloroflexi* | *Dehalococcoidetes* | 205 | 187 | 195 | 91,2 | 95,1 |
| *Bacteria* | *Chloroflexi* | *Elev-1554* | 11 | 7 | 11 | 63,6 | 100,0 |
| *Bacteria* | *Chloroflexi* | *FS117-23B-02* | 16 | 14 | 14 | 87,5 | 87,5 |
| *Bacteria* | *Chloroflexi* | *FS118-62B-02* | 43 | 37 | 42 | 86,0 | 97,7 |
| *Bacteria* | *Chloroflexi* | *FW22* | 32 | 29 | 30 | 90,6 | 93,8 |
| *Bacteria* | *Chloroflexi* | *GIF3* | 46 | 38 | 46 | 82,6 | 100,0 |
| *Bacteria* | *Chloroflexi* | *GIF9* | 86 | 81 | 84 | 94,2 | 97,7 |
| *Bacteria* | *Chloroflexi* | *Gitt-GS-136* | 40 | 39 | 40 | 97,5 | 100,0 |
| *Bacteria* | *Chloroflexi* | *JG30-KF-CM66* | 102 | 99 | 102 | 97,1 | 100,0 |
| *Bacteria* | *Chloroflexi* | *JG37-AG-4* | 127 | 42 | 125 | 33,1 | 98,4 |
| *Bacteria* | *Chloroflexi* | *KD4-96* | 87 | 80 | 84 | 92,0 | 96,6 |
| *Bacteria* | *Chloroflexi* | *KZNMV-5-B42* | 2 | 2 | 2 | 100,0 | 100,0 |
| *Bacteria* | *Chloroflexi* | *Ktedonobacteria* | 376 | 353 | 371 | 93,9 | 98,7 |
| *Bacteria* | *Chloroflexi* | *MSB-5B2* | 8 | 7 | 8 | 87,5 | 100,0 |
| *Bacteria* | *Chloroflexi* | *Napoli-4B-65* | 24 | 22 | 24 | 91,7 | 100,0 |
| *Bacteria* | *Chloroflexi* | *P2-11E* | 28 | 23 | 27 | 82,1 | 96,4 |
| *Bacteria* | *Chloroflexi* | *S085* | 137 | 129 | 134 | 94,2 | 97,8 |
| *Bacteria* | *Chloroflexi* | *SHA-26* | 28 | 25 | 28 | 89,3 | 100,0 |
| *Bacteria* | *Chloroflexi* | *Sh765B-AG-111* | 94 | 93 | 94 | 98,9 | 100,0 |
| *Bacteria* | *Chloroflexi* | *TK10* | 152 | 144 | 150 | 94,7 | 98,7 |
| *Bacteria* | *Chloroflexi* | *Thermomicrobia* | 132 | 129 | 131 | 97,7 | 99,2 |
| *Bacteria* | *Chloroflexi* | *vadinBA26* | 169 | 150 | 166 | 88,8 | 98,2 |
| *Bacteria* | *Chrysiogenetes* | *Chrysiogenetes* | 9 | 7 | 8 | 77,8 | 88,9 |
| *Bacteria* | *Cyanobacteria* | *4C0d-2* | 202 | 188 | 199 | 93,1 | 98,5 |
| *Bacteria* | *Cyanobacteria* | *Acaryochloris* | 15 | 14 | 14 | 93,3 | 93,3 |
| *Bacteria* | *Cyanobacteria* | *Brasilonema* | 12 | 10 | 10 | 83,3 | 83,3 |
| *Bacteria* | *Cyanobacteria* | *Chroogloeocystis* | 4 | 4 | 4 | 100,0 | 100,0 |
| *Bacteria* | *Cyanobacteria* | *ML635J-21* | 100 | 91 | 100 | 91,0 | 100,0 |
| *Bacteria* | *Cyanobacteria* | *MLE1-12* | 78 | 75 | 77 | 96,2 | 98,7 |
| *Bacteria* | *Cyanobacteria* | *Mastigocladopsis* | 7 | 7 | 7 | 100,0 | 100,0 |
| *Bacteria* | *Cyanobacteria* | *Merismopedia* | 16 | 16 | 16 | 100,0 | 100,0 |
| *Bacteria* | *Cyanobacteria* | *QB36* | 2 | 2 | 2 | 100,0 | 100,0 |
| *Bacteria* | *Cyanobacteria* | *SHA-109* | 131 | 117 | 129 | 89,3 | 98,5 |
| *Bacteria* | *Cyanobacteria* | *SM1D11* | 29 | 29 | 29 | 100,0 | 100,0 |
| *Bacteria* | *Cyanobacteria* | *SM2F09* | 11 | 11 | 11 | 100,0 | 100,0 |
| *Bacteria* | *Cyanobacteria* | *Snowella* | 7 | 7 | 7 | 100,0 | 100,0 |
| *Bacteria* | *Cyanobacteria* | *SubsectionI* | 2810 | 2631 | 2724 | 93,6 | 96,9 |
| *Bacteria* | *Cyanobacteria* | *SubsectionII* | 186 | 165 | 177 | 88,7 | 95,2 |
| *Bacteria* | *Cyanobacteria* | *SubsectionIII* | 1770 | 1699 | 1737 | 96,0 | 98,1 |
| *Bacteria* | *Cyanobacteria* | *SubsectionIV* | 1371 | 1319 | 1350 | 96,2 | 98,5 |
| *Bacteria* | *Cyanobacteria* | *SubsectionV* | 101 | 95 | 98 | 94,1 | 97,0 |
| *Bacteria* | *Cyanobacteria* | *WD272* | 54 | 54 | 54 | 100,0 | 100,0 |
| *Bacteria* | *Deferribacteres* | *Deferribacteres* | 1462 | 1403 | 1444 | 96,0 | 98,8 |
| *Bacteria* | *Deinococcus-Thermus* | *Deinococci* | 879 | 811 | 864 | 92,3 | 98,3 |
| *Bacteria* | *Dictyoglomi* | *Dictyoglomia* | 27 | 25 | 26 | 92,6 | 96,3 |
| *Bacteria* | *Elusimicrobia* | *Elusimicrobia* | 312 | 275 | 303 | 88,1 | 97,1 |
| *Bacteria* | *Fibrobacteres* | *Fibrobacteria* | 997 | 930 | 989 | 93,3 | 99,2 |
| *Bacteria* | *Firmicutes* | *Bacillales* | 1 | 0 | 0 | 0,0 | 0,0 |
| *Bacteria* | *Firmicutes* | *Bacilli* | 50612 | 47050 | 49319 | 93,0 | 97,4 |
| *Bacteria* | *Firmicutes* | *Clostridia* | 107605 | 99406 | 105039 | 92,4 | 97,6 |
| *Bacteria* | *Firmicutes* | *Erysipelotrichi* | 7251 | 6151 | 7047 | 84,8 | 97,2 |
| *Bacteria* | *Firmicutes* | *Lactobacillales* | 2 | 0 | 0 | 0,0 | 0,0 |
| *Bacteria* | *Fusobacteria* | *Fusobacteria* | 1706 | 1545 | 1618 | 90,6 | 94,8 |
| *Bacteria* | *Gemmatimonadetes* | *Gemmatimonadetes* | 1461 | 1370 | 1427 | 93,8 | 97,7 |
| *Bacteria* | *Lentisphaerae* | *Lentisphaeria* | 1033 | 466 | 988 | 45,1 | 95,6 |
| *Bacteria* | *Nitrospirae* | *Nitrospira* | 2198 | 2023 | 2139 | 92,0 | 97,3 |
| *Bacteria* | *Planctomycetes* | *028H05-P-BN-P5* | 12 | 10 | 12 | 83,3 | 100,0 |
| *Bacteria* | *Planctomycetes* | *BD7-11* | 59 | 58 | 59 | 98,3 | 100,0 |
| *Bacteria* | *Planctomycetes* | *C47* | 3 | 3 | 3 | 100,0 | 100,0 |
| *Bacteria* | *Planctomycetes* | *MBMPE71* | 13 | 10 | 12 | 76,9 | 92,3 |
| *Bacteria* | *Planctomycetes* | *MD2896-B258* | 7 | 6 | 6 | 85,7 | 85,7 |
| *Bacteria* | *Planctomycetes* | *OM190* | 262 | 196 | 257 | 74,8 | 98,1 |
| *Bacteria* | *Planctomycetes* | *Phycisphaerae* | 1173 | 937 | 1141 | 79,9 | 97,3 |
| *Bacteria* | *Planctomycetes* | *Planctomycetacia* | 3726 | 2884 | 3625 | 77,4 | 97,3 |
| *Bacteria* | *Planctomycetes* | *SGST604* | 2 | 2 | 2 | 100,0 | 100,0 |
| *Bacteria* | *Planctomycetes* | *vadinHA49* | 75 | 60 | 73 | 80,0 | 97,3 |
| *Bacteria* | *Proteobacteria* | *ARKDMS-49* | 17 | 17 | 17 | 100,0 | 100,0 |
| *Bacteria* | *Proteobacteria* | *ARKICE-90* | 48 | 43 | 47 | 89,6 | 97,9 |
| *Bacteria* | *Proteobacteria* | *Alphaproteobacteria* | 46598 | 43100 | 45744 | 92,5 | 98,2 |
| *Bacteria* | *Proteobacteria* | *Betaproteobacteria* | 34843 | 33055 | 34180 | 94,9 | 98,1 |
| *Bacteria* | *Proteobacteria* | *CF2* | 67 | 66 | 67 | 98,5 | 100,0 |
| *Bacteria* | *Proteobacteria* | *Deltaproteobacteria* | 13429 | 10952 | 13144 | 81,6 | 97,9 |
| *Bacteria* | *Proteobacteria* | *Elev-16S-509* | 31 | 27 | 31 | 87,1 | 100,0 |
| *Bacteria* | *Proteobacteria* | *Epsilonproteobacteria* | 4861 | 49 | 4672 | 1,0 | 96,1 |
| *Bacteria* | *Proteobacteria* | *FGL7S* | 6 | 5 | 6 | 83,3 | 100,0 |
| *Bacteria* | *Proteobacteria* | *Gammaproteobacteria* | 89316 | 84731 | 87512 | 94,9 | 98,0 |
| *Bacteria* | *Proteobacteria* | *JTB23* | 122 | 113 | 117 | 92,6 | 95,9 |
| *Bacteria* | *Proteobacteria* | *MACA-EFT26* | 5 | 5 | 5 | 100,0 | 100,0 |
| *Bacteria* | *Proteobacteria* | *Milano-WF1B-44* | 23 | 21 | 23 | 91,3 | 100,0 |
| *Bacteria* | *Proteobacteria* | *SC3-20* | 19 | 16 | 19 | 84,2 | 100,0 |
| *Bacteria* | *Proteobacteria* | *SK259* | 9 | 9 | 9 | 100,0 | 100,0 |
| *Bacteria* | *Proteobacteria* | *SPOTSOCT00m83* | 47 | 44 | 45 | 93,6 | 95,7 |
| *Bacteria* | *Proteobacteria* | *TA18* | 206 | 128 | 198 | 62,1 | 96,1 |
| *Bacteria* | *Proteobacteria* | *pItb-vmat-80* | 4 | 4 | 4 | 100,0 | 100,0 |
| *Bacteria* | *Spirochaetes* | *Spirochaetes* | 4615 | 3990 | 4207 | 86,5 | 91,2 |
| *Bacteria* | *Synergistetes* | *Synergistia* | 1672 | 1595 | 1654 | 95,4 | 98,9 |
| *Bacteria* | *Tenericutes* | *Mollicutes* | 3675 | 903 | 3361 | 24,6 | 91,5 |
| *Bacteria* | *Thermodesulfobacteria* | *Thermodesulfobacteria* | 106 | 99 | 104 | 93,4 | 98,1 |
| *Bacteria* | *Thermotogae* | *Thermotogae* | 736 | 625 | 712 | 84,9 | 96,7 |
| *Bacteria* | *Verrucomicrobia* | *Opitutae* | 521 | 472 | 513 | 90,6 | 98,5 |
| *Bacteria* | *Verrucomicrobia* | *Spartobacteria* | 753 | 30 | 717 | 4,0 | 95,2 |
| *Bacteria* | *Verrucomicrobia* | *Verrucomicrobiae* | 2312 | 42 | 2108 | 1,8 | 91,2 |
|  |  |  |  |  |  |  |  |
| *Eukaryota* |  | *Choanoflagellida* | 1 | 0 | 0 | 0,0 | 0,0 |
| *Eukaryota* | *Alveolata* | *Apicomplexa* | 2037 | 0 | 140 | 0,0 | 6,9 |
| *Eukaryota* | *Alveolata* | *Chromerida* | 3 | 0 | 0 | 0,0 | 0,0 |
| *Eukaryota* | *Alveolata* | *Ciliophora* | 1666 | 0 | 1 | 0,0 | 0,1 |
| *Eukaryota* | *Alveolata* | *Dinophyceae* | 1772 | 0 | 3 | 0,0 | 0,2 |
| *Eukaryota* | *Alveolata* | *Ellobiopsidae* | 6 | 0 | 4 | 0,0 | 66,7 |
| *Eukaryota* | *Alveolata* | *Perkinsea* | 27 | 0 | 0 | 0,0 | 0,0 |
| *Eukaryota* | *Alveolata* | *Voromonas* | 1 | 0 | 0 | 0,0 | 0,0 |
| *Eukaryota* | *Amoebozoa* | *Archamoebae* | 1 | 0 | 0 | 0,0 | 0,0 |
| *Eukaryota* | *Amoebozoa* | *Centramoebida* | 324 | 0 | 0 | 0,0 | 0,0 |
| *Eukaryota* | *Amoebozoa* | *Flabellinea* | 47 | 0 | 24 | 0,0 | 51,1 |
| *Eukaryota* | *Amoebozoa* | *Mycetozoa* | 225 | 0 | 3 | 0,0 | 1,3 |
| *Eukaryota* | *Amoebozoa* | *Phalansterium* | 1 | 0 | 0 | 0,0 | 0,0 |
| *Eukaryota* | *Amoebozoa* | *Tubulinea* | 69 | 0 | 0 | 0,0 | 0,0 |
| *Eukaryota* | *Apusozoa* | *Ancyromonadidae* | 18 | 0 | 0 | 0,0 | 0,0 |
| *Eukaryota* | *Apusozoa* | *Apusomonadidae* | 18 | 0 | 0 | 0,0 | 0,0 |
| *Eukaryota* | *Breviata* |  | 1 | 0 | 0 | 0,0 | 0,0 |
| *Eukaryota* | *Carpediemonas* |  | 4 | 0 | 0 | 0,0 | 0,0 |
| *Eukaryota* | *Centroheliozoa* | *Acanthocystidae* | 18 | 0 | 0 | 0,0 | 0,0 |
| *Eukaryota* | *Centroheliozoa* | *Heterophryidae* | 4 | 0 | 0 | 0,0 | 0,0 |
| *Eukaryota* | *Choanoflagellida* | *Acanthoecidae* | 11 | 0 | 0 | 0,0 | 0,0 |
| *Eukaryota* | *Choanoflagellida* | *Codonosigidae* | 15 | 0 | 0 | 0,0 | 0,0 |
| *Eukaryota* | *Choanoflagellida* | *Salpingoecidae* | 16 | 0 | 0 | 0,0 | 0,0 |
| *Eukaryota* | *Corallochytrium* |  | 1 | 0 | 0 | 0,0 | 0,0 |
| *Eukaryota* | *Cryptophyta* | *Cryptomonadales* | 127 | 0 | 0 | 0,0 | 0,0 |
| *Eukaryota* | *Cryptophyta* | *Pyrenomonadales* | 102 | 0 | 0 | 0,0 | 0,0 |
| *Eukaryota* | *Dimorpha* |  | 1 | 0 | 0 | 0,0 | 0,0 |
| *Eukaryota* | *Diplomonadida* | *Enteromonadidae* | 2 | 0 | 0 | 0,0 | 0,0 |
| *Eukaryota* | *Diplomonadida* | *Hexamitidae* | 35 | 0 | 0 | 0,0 | 0,0 |
| *Eukaryota* | *Dysnectes* |  | 2 | 0 | 0 | 0,0 | 0,0 |
| *Eukaryota* | *Eccrinales* | *Eccrinaceae* | 10 | 0 | 0 | 0,0 | 0,0 |
| *Eukaryota* | *Eccrinales* | *Palavasciaceae* | 1 | 0 | 0 | 0,0 | 0,0 |
| *Eukaryota* | *Ergobibamus* |  | 1 | 0 | 0 | 0,0 | 0,0 |
| *Eukaryota* | *Euglenozoa* | *Diplonemida* | 61 | 0 | 0 | 0,0 | 0,0 |
| *Eukaryota* | *Euglenozoa* | *Euglenida* | 341 | 0 | 14 | 0,0 | 4,1 |
| *Eukaryota* | *Euglenozoa* | *Kinetoplastida* | 595 | 0 | 0 | 0,0 | 0,0 |
| *Eukaryota* | *Fungi* |  | 86 | 0 | 0 | 0,0 | 0,0 |
| *Eukaryota* | *Fungi* | *Blastocladiomycota* | 27 | 0 | 0 | 0,0 | 0,0 |
| *Eukaryota* | *Fungi* | *Chytridiomycota* | 474 | 0 | 2 | 0,0 | 0,4 |
| *Eukaryota* | *Fungi* | *Dikarya* | 9030 | 0 | 15 | 0,0 | 0,2 |
| *Eukaryota* | *Fungi* | *Glomeromycota* | 679 | 0 | 1 | 0,0 | 0,1 |
| *Eukaryota* | *Fungi* | *Microsporidia* | 75 | 0 | 0 | 0,0 | 0,0 |
| *Eukaryota* | *Fungi* | *Neocallimastigomycota* | 17 | 0 | 0 | 0,0 | 0,0 |
| *Eukaryota* | *Fungi* | *cryptomycota* | 2 | 0 | 0 | 0,0 | 0,0 |
| *Eukaryota* | *Glaucocystophyceae* | *Cyanophoraceae* | 2 | 0 | 0 | 0,0 | 0,0 |
| *Eukaryota* | *Glaucocystophyceae* | *Cyanoptyche* | 1 | 0 | 0 | 0,0 | 0,0 |
| *Eukaryota* | *Glaucocystophyceae* | *Glaucocystales* | 1 | 0 | 0 | 0,0 | 0,0 |
| *Eukaryota* | *Glaucocystophyceae* | *Gloeochaetales* | 1 | 0 | 0 | 0,0 | 0,0 |
| *Eukaryota* | *Haptophyceae* | *Coccolithales* | 32 | 0 | 0 | 0,0 | 0,0 |
| *Eukaryota* | *Haptophyceae* | *Coccosphaerales* | 8 | 0 | 0 | 0,0 | 0,0 |
| *Eukaryota* | *Haptophyceae* | *Isochrysidales* | 39 | 0 | 0 | 0,0 | 0,0 |
| *Eukaryota* | *Haptophyceae* | *Pavlovales* | 54 | 0 | 0 | 0,0 | 0,0 |
| *Eukaryota* | *Haptophyceae* | *Phaeocystales* | 33 | 0 | 0 | 0,0 | 0,0 |
| *Eukaryota* | *Haptophyceae* | *Prymnesiales* | 83 | 0 | 0 | 0,0 | 0,0 |
| *Eukaryota* | *Haptophyceae* | *Reticulosphaerales* | 1 | 0 | 0 | 0,0 | 0,0 |
| *Eukaryota* | *Haptophyceae* | *Syracosphaerales* | 3 | 0 | 0 | 0,0 | 0,0 |
| *Eukaryota* | *Haptophyceae* | *Zygodiscales* | 6 | 0 | 0 | 0,0 | 0,0 |
| *Eukaryota* | *Heterolobosea* |  | 2 | 0 | 0 | 0,0 | 0,0 |
| *Eukaryota* | *Heterolobosea* | *Acrasida* | 1 | 0 | 0 | 0,0 | 0,0 |
| *Eukaryota* | *Heterolobosea* | *Learamoeba* | 1 | 0 | 0 | 0,0 | 0,0 |
| *Eukaryota* | *Heterolobosea* | *Monopylocystis* | 1 | 0 | 0 | 0,0 | 0,0 |
| *Eukaryota* | *Heterolobosea* | *Schizopyrenida* | 66 | 0 | 0 | 0,0 | 0,0 |
| *Eukaryota* | *Heterolobosea* | *Singhamoeba* | 2 | 0 | 0 | 0,0 | 0,0 |
| *Eukaryota* | *Heterolobosea* | *Stephanopogon* | 1 | 0 | 0 | 0,0 | 0,0 |
| *Eukaryota* | *Heterolobosea* | *Tulamoeba* | 1 | 0 | 0 | 0,0 | 0,0 |
| *Eukaryota* | *Hicanonectes* |  | 1 | 0 | 0 | 0,0 | 0,0 |
| *Eukaryota* | *Ichthyosporea* | *Capsaspora* | 9 | 0 | 0 | 0,0 | 0,0 |
| *Eukaryota* | *Ichthyosporea* | *Dermocystida* | 24 | 0 | 0 | 0,0 | 0,0 |
| *Eukaryota* | *Ichthyosporea* | *Ichthyophonida* | 58 | 0 | 0 | 0,0 | 0,0 |
| *Eukaryota* | *Jakobida* | *Andalucia* | 4 | 0 | 0 | 0,0 | 0,0 |
| *Eukaryota* | *Jakobida* | *Jakobidae* | 2 | 0 | 0 | 0,0 | 0,0 |
| *Eukaryota* | *Katablepharidophyta* | *Katablepharidaceae* | 14 | 0 | 0 | 0,0 | 0,0 |
| *Eukaryota* | *Katablepharidophyta* | *Roombia* | 1 | 0 | 0 | 0,0 | 0,0 |
| *Eukaryota* | *Kipferlia* |  | 11 | 0 | 0 | 0,0 | 0,0 |
| *Eukaryota* | *Malawimonadidae* | *Malawimonas* | 2 | 0 | 0 | 0,0 | 0,0 |
| *Eukaryota* | *Metazoa* | *Acanthocephala* | 51 | 0 | 46 | 0,0 | 90,2 |
| *Eukaryota* | *Metazoa* | *Annelida* | 1400 | 0 | 0 | 0,0 | 0,0 |
| *Eukaryota* | *Metazoa* | *Arthropoda* | 10189 | 0 | 56 | 0,0 | 0,5 |
| *Eukaryota* | *Metazoa* | *Brachiopoda* | 79 | 0 | 0 | 0,0 | 0,0 |
| *Eukaryota* | *Metazoa* | *Bryozoa* | 97 | 0 | 0 | 0,0 | 0,0 |
| *Eukaryota* | *Metazoa* | *Chaetognatha* | 22 | 0 | 1 | 0,0 | 4,5 |
| *Eukaryota* | *Metazoa* | *Chordata* | 1063 | 0 | 0 | 0,0 | 0,0 |
| *Eukaryota* | *Metazoa* | *Cnidaria* | 1462 | 0 | 14 | 0,0 | 1,0 |
| *Eukaryota* | *Metazoa* | *Ctenophora* | 34 | 0 | 0 | 0,0 | 0,0 |
| *Eukaryota* | *Metazoa* | *Cycliophora* | 19 | 0 | 0 | 0,0 | 0,0 |
| *Eukaryota* | *Metazoa* | *Echinodermata* | 228 | 0 | 1 | 0,0 | 0,4 |
| *Eukaryota* | *Metazoa* | *Echiura* | 6 | 0 | 0 | 0,0 | 0,0 |
| *Eukaryota* | *Metazoa* | *Entoprocta* | 18 | 0 | 0 | 0,0 | 0,0 |
| *Eukaryota* | *Metazoa* | *Gastrotricha* | 29 | 0 | 1 | 0,0 | 3,4 |
| *Eukaryota* | *Metazoa* | *Hemichordata* | 26 | 0 | 0 | 0,0 | 0,0 |
| *Eukaryota* | *Metazoa* | *Kinorhyncha* | 9 | 0 | 0 | 0,0 | 0,0 |
| *Eukaryota* | *Metazoa* | *Loricifera* | 1 | 0 | 0 | 0,0 | 0,0 |
| *Eukaryota* | *Metazoa* | *Mesozoa* | 6 | 0 | 0 | 0,0 | 0,0 |
| *Eukaryota* | *Metazoa* | *Micrognathozoa* | 2 | 0 | 0 | 0,0 | 0,0 |
| *Eukaryota* | *Metazoa* | *Mollusca* | 1489 | 0 | 2 | 0,0 | 0,1 |
| *Eukaryota* | *Metazoa* | *Myzostomida* | 45 | 0 | 0 | 0,0 | 0,0 |
| *Eukaryota* | *Metazoa* | *Nematoda* | 2146 | 0 | 3 | 0,0 | 0,1 |
| *Eukaryota* | *Metazoa* | *Nematomorpha* | 19 | 0 | 0 | 0,0 | 0,0 |
| *Eukaryota* | *Metazoa* | *Nemertea* | 102 | 0 | 0 | 0,0 | 0,0 |
| *Eukaryota* | *Metazoa* | *Onychophora* | 14 | 0 | 0 | 0,0 | 0,0 |
| *Eukaryota* | *Metazoa* | *Placozoa* | 12 | 0 | 0 | 0,0 | 0,0 |
| *Eukaryota* | *Metazoa* | *Platyhelminthes* | 1422 | 0 | 9 | 0,0 | 0,6 |
| *Eukaryota* | *Metazoa* | *Porifera* | 300 | 0 | 1 | 0,0 | 0,3 |
| *Eukaryota* | *Metazoa* | *Priapulida* | 23 | 0 | 0 | 0,0 | 0,0 |
| *Eukaryota* | *Metazoa* | *Rotifera* | 120 | 0 | 0 | 0,0 | 0,0 |
| *Eukaryota* | *Metazoa* | *Sipuncula* | 72 | 0 | 0 | 0,0 | 0,0 |
| *Eukaryota* | *Metazoa* | *Tardigrada* | 214 | 0 | 0 | 0,0 | 0,0 |
| *Eukaryota* | *Metazoa* | *Xenoturbellida* | 2 | 0 | 0 | 0,0 | 0,0 |
| *Eukaryota* | *Metromonas* |  | 1 | 0 | 0 | 0,0 | 0,0 |
| *Eukaryota* | *Micronuclearia* |  | 1 | 0 | 0 | 0,0 | 0,0 |
| *Eukaryota* | *Ministeria* |  | 2 | 0 | 0 | 0,0 | 0,0 |
| *Eukaryota* | *Parabasalia* |  | 48 | 0 | 0 | 0,0 | 0,0 |
| *Eukaryota* | *Parabasalia* | *Cristamonadida* | 61 | 0 | 0 | 0,0 | 0,0 |
| *Eukaryota* | *Parabasalia* | *Honigbergiellida* | 9 | 0 | 0 | 0,0 | 0,0 |
| *Eukaryota* | *Parabasalia* | *Hypotrichomonadida* | 4 | 0 | 0 | 0,0 | 0,0 |
| *Eukaryota* | *Parabasalia* | *Spirotrichonymphida* | 2 | 0 | 0 | 0,0 | 0,0 |
| *Eukaryota* | *Parabasalia* | *Trichomonadida* | 106 | 0 | 0 | 0,0 | 0,0 |
| *Eukaryota* | *Parabasalia* | *Trichonymphida* | 84 | 0 | 0 | 0,0 | 0,0 |
| *Eukaryota* | *Parabasalia* | *Tritrichomonadida* | 42 | 0 | 0 | 0,0 | 0,0 |
| *Eukaryota* | *Proleptomonas* |  | 3 | 0 | 0 | 0,0 | 0,0 |
| *Eukaryota* | *Reticulamoeba* |  | 3 | 0 | 0 | 0,0 | 0,0 |
| *Eukaryota* | *Rhizaria* | *Acantharea* | 27 | 0 | 26 | 0,0 | 96,3 |
| *Eukaryota* | *Rhizaria* | *Cercozoa* | 817 | 0 | 6 | 0,0 | 0,7 |
| *Eukaryota* | *Rhizaria* | *Foraminifera* | 103 | 0 | 0 | 0,0 | 0,0 |
| *Eukaryota* | *Rhizaria* | *Gromiidae* | 3 | 0 | 0 | 0,0 | 0,0 |
| *Eukaryota* | *Rhizaria* | *Haplosporidia* | 35 | 0 | 2 | 0,0 | 5,7 |
| *Eukaryota* | *Rhizaria* | *Polycystinea* | 70 | 0 | 9 | 0,0 | 12,9 |
| *Eukaryota* | *Rhizaria* | *Sticholonche* | 2 | 0 | 2 | 0,0 | 100,0 |
| *Eukaryota* | *Rhodophyta* | *Bangiophyceae* | 388 | 0 | 1 | 0,0 | 0,3 |
| *Eukaryota* | *Rhodophyta* | *Compsopogonophyceae* | 23 | 0 | 0 | 0,0 | 0,0 |
| *Eukaryota* | *Rhodophyta* | *Florideophyceae* | 1104 | 0 | 124 | 0,0 | 11,2 |
| *Eukaryota* | *Rhodophyta* | *Rhodellophyceae* | 18 | 0 | 1 | 0,0 | 5,6 |
| *Eukaryota* | *Rhodophyta* | *Stylonematophyceae* | 13 | 0 | 0 | 0,0 | 0,0 |
| *Eukaryota* | *Telonemida* | *Telonema* | 11 | 0 | 9 | 0,0 | 81,8 |
| *Eukaryota* | *Trimastix* |  | 3 | 0 | 0 | 0,0 | 0,0 |
| *Eukaryota* | *Viridiplantae* | *Chlorophyta* | 2477 | 0 | 2 | 0,0 | 0,1 |
| *Eukaryota* | *Viridiplantae* | *Streptophyta* | 5119 | 0 | 6 | 0,0 | 0,1 |
| *Eukaryota* | *stramenopiles* |  | 24 | 0 | 0 | 0,0 | 0,0 |
| *Eukaryota* | *stramenopiles* | *Bacillariophyta* | 1302 | 0 | 0 | 0,0 | 0,0 |
| *Eukaryota* | *stramenopiles* | *Bicosoecida* | 60 | 0 | 0 | 0,0 | 0,0 |
| *Eukaryota* | *stramenopiles* | *Blastocystis* | 159 | 0 | 0 | 0,0 | 0,0 |
| *Eukaryota* | *stramenopiles* | *Bolidophyceae* | 10 | 0 | 0 | 0,0 | 0,0 |
| *Eukaryota* | *stramenopiles* | *Chrysomerophyceae* | 2 | 0 | 0 | 0,0 | 0,0 |
| *Eukaryota* | *stramenopiles* | *Chrysophyceae* | 295 | 0 | 0 | 0,0 | 0,0 |
| *Eukaryota* | *stramenopiles* | *Developayella* | 1 | 0 | 0 | 0,0 | 0,0 |
| *Eukaryota* | *stramenopiles* | *Dictyochophyceae* | 31 | 0 | 0 | 0,0 | 0,0 |
| *Eukaryota* | *stramenopiles* | *Eustigmatophyceae* | 90 | 0 | 0 | 0,0 | 0,0 |
| *Eukaryota* | *stramenopiles* | *Hyphochytriomycetes* | 3 | 0 | 0 | 0,0 | 0,0 |
| *Eukaryota* | *stramenopiles* | *Labyrinthulida* | 329 | 0 | 0 | 0,0 | 0,0 |
| *Eukaryota* | *stramenopiles* | *Oikomonadaceae* | 3 | 0 | 0 | 0,0 | 0,0 |
| *Eukaryota* | *stramenopiles* | *Olisthodiscus* | 1 | 0 | 0 | 0,0 | 0,0 |
| *Eukaryota* | *stramenopiles* | *Oomycetes* | 242 | 0 | 0 | 0,0 | 0,0 |
| *Eukaryota* | *stramenopiles* | *Pelagophyceae* | 28 | 0 | 0 | 0,0 | 0,0 |
| *Eukaryota* | *stramenopiles* | *Phaeothamniophyceae* | 9 | 0 | 0 | 0,0 | 0,0 |
| *Eukaryota* | *stramenopiles* | *Pinguiophyceae* | 9 | 0 | 0 | 0,0 | 0,0 |
| *Eukaryota* | *stramenopiles* | *Pirsonia* | 7 | 0 | 0 | 0,0 | 0,0 |
| *Eukaryota* | *stramenopiles* | *Placididea* | 2 | 0 | 0 | 0,0 | 0,0 |
| *Eukaryota* | *stramenopiles* | *Pseudopirsonia* | 1 | 0 | 0 | 0,0 | 0,0 |
| *Eukaryota* | *stramenopiles* | *Raphidophyceae* | 53 | 0 | 0 | 0,0 | 0,0 |
| *Eukaryota* | *stramenopiles* | *Schizocladia* | 1 | 0 | 0 | 0,0 | 0,0 |
| *Eukaryota* | *stramenopiles* | *Slopalinida* | 6 | 0 | 0 | 0,0 | 0,0 |
| *Eukaryota* | *stramenopiles* | *Solenicola* | 3 | 0 | 0 | 0,0 | 0,0 |
| *Eukaryota* | *stramenopiles* | *Synchromophyceae* | 1 | 0 | 0 | 0,0 | 0,0 |
| *Eukaryota* | *stramenopiles* | *Synurophyceae* | 214 | 0 | 0 | 0,0 | 0,0 |
